# Supplementary material for: The Spatiotemporal Characteristics of 0–24-Goal Polo
Source: Animals (Basel). 2019 Jul 16;9(7):446. doi: 10.3390/ani9070446 (PMC6680633; doi:10.3390/ani9070446)
Supplement: Supplementary file 1 [file animals-09-00446-s001.pdf]

Table S1. Effect sizes ± 90 % Confidence Intervals for mean distance (m) per chukka, compared at each level of play. Values are presented in **bold** if confidence interval does not cross zero. Magnitude of the effect is denoted by the following symbols *: Small; # Moderate; †: Large; ‡: Very Large and is only provided for those effects whose confidence intervals do not cross zero.

Comparison	Speed Zone 1			Speed Zone 2			Speed Zone 3			Speed Zone 4			Speed Zone 5		
0 goal – 6 goal	**-0.44**	**±**	**0.36***	**0.42**	**±**	**0.36***	0.17	±	0.36	**-0.46**	**±**	**0.37***	**-0.49**	**±**	**0.49***
0 goal – 10 goal	-0.03	±	0.31	-0.03	±	0.31	-0.07	±	0.32	**-0.74**	**±**	**0.33#**	**-0.58**	**±**	**0.31***
0 goal – 16 goal	**-0.97**	**±**	**0.30#**	**1.05**	**±**	**0.30#**	**0.60**	**±**	**0.29#**	**-1.40**	**±**	**0.31†**	**-0.83**	**±**	**0.30#**
0 goal – 24 goal	**-0.77**	**±**	**0.43#**	-0.23	±	0.42	**-0.65**	**±**	**0.43#**	**-2.05**	**±**	**0.51‡**	**-2.10**	**±**	**0.52‡**
6 goal – 10 goal	0.26	±	0.31	**-0.56**	**±**	**0.32***	**-0.33**	**±**	**0.32***	-0.27	±	0.31	-0.08	±	0.31
6 goal – 16 goal	**-0.85**	**±**	**0.30#**	**0.74**	**±**	**0.30#**	**0.47**	**±**	**0.29***	**-1.03**	**±**	**0.30#**	**-0.51**	**±**	**0.29***
6 goal – 24 goal	**-0.79**	**±**	**0.43#**	**-0.71**	**±**	**0.44#**	**-1.07**	**±**	**0.45#**	**-1.53**	**±**	**0.47†**	**-1.36**	**±**	**0.47†**
10 goal – 16 goal	**-1.00**	**±**	**0.24#**	**-1.18**	**±**	**0.24#**	**0.78**	**±**	**0.24***	**-0.86**	**±**	**0.23***	**-0.49**	**±**	**0.22***
10 goal – 24 goal	**-0.59**	**±**	**0.39***	-0.26	±	0.38	**-0.95**	**±**	**0.40#**	**-1.35**	**±**	**0.42†**	**-1.47**	**±**	**0.41†**
16 goal – 24 goal	**0.59**	**±**	**0.37***	**-1.23**	**±**	**0.37†**	**-1.38**	**±**	**0.38†**	-0.24	±	0.37	**-0.64**	**±**	**0.36#**

Table S2. Effect sizes ± 90 % Confidence Intervals for mean time (min:s) in speed zone per chukka, compared at each level of play. Values are presented in **bold** if confidence interval does not cross zero. Magnitude of the effect is denoted by the following symbols *: Small; # Moderate; †: Large; ‡: Very Large and is only provided for those effects whose confidence intervals do not cross zero.

Comparison	Speed Zone 1			Speed Zone 2			Speed Zone 3			Speed Zone 4			Speed Zone 5		
0 goal – 6 goal	0.06	±	0.35	**0.39**	**±**	**0.36***	0.22	±	0.36	**-0.46**	**±**	**0.36***	**-0.49**	**±**	**0.36***
0 goal – 10 goal	**0.37**	**±**	**0.31***	-0.05	±	0.31	-0.05	±	0.31	**-0.74**	**±**	**0.32#**	**-0.58**	**±**	**0.32***
0 goal – 16 goal	-0.09	±	0.28	**1.06**	**±**	**0.30#**	**0.60**	**±**	**0.29#**	**-1.39**	**±**	**0.31†**	**-0.84**	**±**	**0.30#**
0 goal – 24 goal	-0.16	±	0.42	-0.17	±	0.42	**-0.62**	**±**	**0.43#**	**-2.01**	**±**	**0.51‡**	**-2.17**	**±**	**0.59‡**
6 goal – 10 goal	-0.30	±	0.31	**-0.54**	**±**	**0.31***	**-0.37**	**±**	**0.32***	-0.27	±	0.32	-0.08	±	0.31
6 goal – 16 goal	-0.14	±	0.29	**0.79**	**±**	**0.29#**	**0.42**	**±**	**0.29***	**-1.08**	**±**	**0.30#**	**-0.52**	**±**	**0.29***
6 goal – 24 goal	-0.22	±	0.42	**-0.63**	**±**	**0.43#**	**-1.08**	**±**	**0.45#**	**-1.51**	**±**	**0.47†**	**-1.40**	**±**	**0.46†**
10 goal – 16 goal	**-0.43**	**±**	**0.23***	**1.20**	**±**	**0.24†**	**0.76**	**±**	**0.23#**	**-0.94**	**±**	**0.23#**	**-0.50**	**±**	**0.22***
10 goal – 24 goal	**-0.58**	**±**	**0.39***	-0.17	±	0.39	**-0.92**	**±**	**0.39#**	**-1.33**	**±**	**0.41†**	**-1.51**	**±**	**0.42†**
16 goal – 24 goal	-0.06	±	0.36	**-1.19**	**±**	**0.37#**	**-1.37**	**±**	**0.39†**	-0.03	±	0.36	**-0.65**	**±**	**0.36#**

Table S3. Effect sizes ± 90 % Confidence Intervals for mean high intensity activities per chukka, compared at each level of play. Values are presented in **bold** if confidence interval does not cross zero. Magnitude of the effect is denoted by the following symbols *: Small; # Moderate; †: Large; ‡: Very Large and is only provided for those effects whose confidence intervals do not cross zero.

Comparison	Sprints			Accelerations			Decelerations			Impacts		
0 goal – 6 goal	0.33	±	0.36	0.17	±	0.36	0.01	±	0.36	-0.32	±	0.36
0 goal – 10 goal	-0.18	±	0.31	0.30	±	0.31	**-0.39**	**±**	**0.32***	-0.24	±	0.31
0 goal – 16 goal	**-0.49**	**±**	**0.29***	**-0.72**	**±**	**0.72#**	**-0.81**	**±**	**0.30#**	**-0.49**	**±**	**0.29***
0 goal – 24 goal	**-0.90**	**±**	**0.44#**	-0.09	±	0.43	**-1.18**	**±**	**0.45#**	**-0.97**	**±**	**0.44#**
6 goal – 10 goal	**-0.55**	**±**	**0.31***	0.08	±	0.32	**-0.37**	**±**	**0.32***	0.17	±	0.32
6 goal – 16 goal	**-0.85**	**±**	**0.30#**	**-0.94**	**±**	**0.30#**	**-0.80**	**±**	**0.29#**	-0.23	±	0.29
6 goal – 24 goal	**-1.24**	**±**	**0.23†**	-0.29	±	0.43	**-1.10**	**±**	**0.45#**	-0.28	±	0.42
10 goal – 16 goal	**-0.32**	**±**	**0.23***	**-1.13**	**±**	**0.24#**	**-0.53**	**±**	**0.23***	**-0.39**	**±**	**0.23***
10 goal – 24 goal	**-0.83**	**±**	**0.40#**	**-0.50**	**±**	**0.39***	**-0.99**	**±**	**0.40#**	**-0.64**	**±**	**0.35#**
16 goal – 24 goal	**-0.49**	**±**	**0.37***	**0.68**	**±**	**0.37#**	-0.33	±	0.36	0.00	±	0.36
